# Tumor endothelial cell-derived Sfrp1 supports the maintenance of cancer stem cells via Wnt signaling

**DOI:** 10.1007/s11626-024-00899-y

**Published:** 2024-04-16

**Authors:** Yumiko Hayashi, Masakazu Hashimoto, Katsuyoshi Takaoka, Tatsuya Takemoto, Nobuyuki Takakura, Hiroyasu Kidoya

**Affiliations:** 1 Department of Integrative Vascular Biology, Faculty of Medical Science, Fukui University, 23-3 Matsuoka-Shimoaizuki, Eiheiji, Yoshida, Fukui 910-1193 Japan; 2 Department of Signal Transduction, Research Institute for Microbial Diseases, Osaka University, Suita, Japan; 3 Laboratory for Embryogenesis, Graduate School of Frontier Biosciences, Osaka University, Suita, Osaka Japan; 4 Laboratory for Embryology, Institute of Advanced Medical Sciences, Tokushima University, Tokushima, Japan; 5 World Premier Institute Immunology Frontier Research Center, Integrated Frontier Research for Medical Science Division, Osaka University, Suita, Japan; 6 Institute for Open and Transdisciplinary Research Initiatives (OTRI), Osaka University, Suita, Japan; 7 Center for Infectious Disease Education and Research, Osaka University, Suita, Japan

**Keywords:** Tumor endothelial cell, Angiocrine factor, Sfrp1, Cancer stem cell, Wnt signaling

## Abstract

Cancer stem cells (CSCs), which are critical targets for cancer therapy as they are involved in drug resistance to anticancer drugs, and metastasis, are maintained by angiocrine factors produced by particular niches that form within tumor tissue. Secreted frizzled-related protein 1 (Sfrp1) is an extracellular protein that modulates Wnt signaling. However, the cells that produce Sfrp1 in the tumor environment and its function remain unclear. We aimed to elucidate angiocrine factors related to CSC maintenance, focusing on Sfrp1. Although Sfrp1 is a Wnt pathway-related factor, its impact on tumor tissues remains unknown. We investigated the localization of Sfrp1 in tumors and found that it is expressed in some tumor vessels. Analysis of mice lacking Sfrp1 showed that tumor growth was suppressed in Sfrp1-deficient tumor tissues. Flow cytometry analysis indicated that CSCs were maintained in the early tumor growth phase in the Sfrp1 knockout (KO) mouse model of tumor-bearing cancer. However, tumor growth was inhibited in the late tumor growth phase because of the inability to maintain CSCs. Real-time PCR results from tumors of Sfrp1 KO mice showed that the expression of Wnt signaling target genes significantly decreased in the late stage of tumor growth. This suggests that Sfrp1, an angiocrine factor produced by the tumor vascular niche, is involved in Wnt signaling-mediated mechanisms in tumor tissues.

**Supplementary Information:**

The online version contains supplementary material available at 10.1007/s11626-024-00899-y.

## Introduction

The vascular network is not only responsible for the transport of oxygen and nutrients but also plays a critical role in organogenesis and homeostasis through the production of angiocrine factors. Angiocrine factors are secreted molecules expressed by vascular endothelial cells (ECs) and other components of blood vessels that act on cell populations in the tissue microenvironment (Rafii *et al*. 2016). Blood vessels that produce angiocrine factors are called vascular niches and regulate the differentiation and proliferation of stem cells, including neural and hematopoietic stem cells, in various tissues (Ramasamy *et al*. 2016; Karakatsani *et al*. 2023). In cancer tissues, blood vessels form a niche that contributes to the maintenance of cancer stem cells and regulates their proliferation (Alsina-Sanchis *et al*. 2021). Therefore, the tumor vascular niche and angiocrine factors are potential targets for cancer therapies. CSCs in tumor tissues represent a highly malignant, specialized population of cancer cells (CCs). The frequencies of metastasis and recurrence, which determine the malignant potential of cancer, are largely dependent on the degree of CSC control (Batlle and Clevers 2017; Atkins *et al*. 2019). Tumor blood vessels in tumor tissue not only supply nutrients to CCs but also form a niche in which CSCs are maintained (Krishnamurthy *et al*. 2010; Hira *et al*. 2015). Direct targeting of CSCs is challenging because of their drug resistance (Ishimoto *et al*. 2011). Therefore, there is a need to develop indirect therapies targeting the CSC niche.

Secreted frizzled-related protein 1 is an extracellular protein that modulates Wnt signaling. The Wnt pathway is involved in cell proliferation, differentiation, anchoring, apoptosis, and cell cycle regulation (Clevers 2006; Niehrs 2012; Skronska-Wasek *et al*. 2017; Perugorria *et al*. 2019). The Sfrp1 protein harbors two structural domains: a carboxy-terminal netrin (NTR) domain and an amino-terminal cysteine-rich domain (CRD). It has been postulated that Sfrp1 serves as an antagonist of Wnt because the CRD of Sfrp1 is similar to that of the frizzled receptor (Finch *et al*. 1997; Bhat *et al*. 2007). However, Sfrp1 has been shown to directly activate intracellular signaling by binding to Frizzled receptors and may modulate the Wnt signaling cascade in positive and negative ways in a context-dependent manner (Rodriguez *et al*. 2005). The expression of Sfrp1 is downregulated in cancer tissues and is presumed to function in cancer suppression by dysregulating cell proliferation and invasion (Atschekzei *et al*. 2012). However, the cells that produce Sfrp1 in the tumor microenvironment and the function of Sfrp1 remain underexplored. In this study, we clarified the effects of Sfrp1 on tumor tissues and analyzed its interactions with tumor blood vessels.

## Materials and methods

### Generation of Sfrp1^−/−^ mice using CRISPR-Cas9 technology

C57BL/6N mice were purchased from Japan SLC (Shizuoka, Japan) and Sankyo Labo Service Corporation (Toyama, Japan). Sfrp1 knockout mice were generated using the CRISPR-Cas9 genome editing system (Hashimoto *et al*. 2016). C57BL/6N female mice were superovulated by intraperitoneal injection of pregnant gonadotropin, and after 48 h, human chorionic gonadotropin. One-cell-stage zygotes were collected from the oviduct ampulla in M2 medium, followed by injection of a combination of single guide (sgRNA) and Cas9 mRNA through electroporation. These were then implanted into the oviducts of pseudopregnant females. The sgRNA target sequences for *Sfrp1* are as follows: gRNA1, 5′-ACA TCG GCT CGT ATC AGA GC-3′; gRNA2, 5′-CTG AGG CTG TGC CAC AAC GT-3′; and gRNA3, 5′-CAA ATG TGA CAA GTT CCC CG-3′. The animals were housed in environmentally controlled rooms of animal experimentation facilities at Osaka and Fukui Universities. All experiments were carried out according to the Osaka and Fukui University Committee for Animals guidelines and approved by the Osaka and Fukui University Institutional Review Boards.

### Genotype identification

Sfrp1^−/−^ mutation was identified via Sanger sequencing. Polymerase chain reaction and Sanger sequencing were performed using the following primers: forward primer1, 5′-GCC AGC GAG TAC GAC TAC GTG AG-3′; reverse primer1, 5′-CCA AGG TAA GGG TAT GCC TTC CCA-3′ and forward primer2, 5′-AGG ACC CCA TCG ATC GGA GAC-3′; reverse primer2, 5′-CCA GTC TGG CGT TTT CCA TAC CTG-3′.

### Cell lines, plasmid construction, and transfection

Cell lines, including Lewis lung carcinoma (LLC) and MC38, were purchased from the Riken BRC Cell Bank (Tsukuba, Japan). Both cell lines were cultured in Dulbecco’s modified Eagle’s medium (Sigma, St. Louis, MO) containing 10% fetal bovine serum (FBS; Gibco, Grand Island, NY) and 1% penicillin/streptomycin (100 U/mL, PS; Life Technologies, Tokyo, Japan). Full-length Sfrp1 cDNA was isolated from total mouse RNA using PCR-based cloning methods, as described previously (Hayashi *et al*. 2019). Primers used for cloning were as follows: sense, 5′-TCT ATC CGA ATT CAG CAA CAT GGG CGT CGG GCG-3′ and anti-sense, 5′-TCT ATC CGT CGA CTC ACT TAA AAA CAG ACT GGA-3′. The PCR product containing the EcoRI/SalI fragment was inserted into the pIRES-EGFP vector (Clontech, Mountain View, CA). LLC cells were transfected with pCMV-mSfrp1-IRES2-EGFP containing mouse Sfrp1 or a mock vector as a control. According to the manufacturer’s instructions, all cells were stably transfected using Lipofectamine 2000 (Invitrogen, Carlsbad, CA). GFP^+^ cells were selected based on antibiotic resistance to G418 (Geneticin; Gibco) by addition to the culture medium and sorted using flow cytometry (FACSAria, BD, San Jose, CA).

### Tumor transplantation model

LLC or MC38 cells (1 × 10^6^ cells per mouse in 100 µL phosphate-buffered saline [PBS]) were inoculated subcutaneously into wild-type (WT; C57BL/6) or Sfrp1^−/−^ mice (8–9 wk of age). Tumor volumes were measured using calipers every 2–3 d and calculated as follows: length × width × width × 0.52.

### Immunostaining analysis

Tumors were fixed using 4% paraformaldehyde in PBS, treated with 15% sucrose in PBS, followed by 30% sucrose in PBS, and embedded in an optimal cutting temperature compound (Sakura Finetek, Tokyo, Japan). Frozen blocks were sectioned into slices measuring 20 or 40 µm. The following primary antibodies (Abs) were used: rat anti-mouse CD31 Ab (BD Bioscience, Franklin Lakes, NY), hamster anti-mouse CD31 Ab (Merck Millipore, Darmstadt, Germany), rabbit anti-mouse Sfrp1 Ab (Sigma), and αSMA-Cy3 (Merck Millipore). Alexa Fluor 488-conjugated anti-rat IgG (Invitrogen), Alexa Fluor 488-conjugated anti-hamster IgG (Jackson ImmunoResearch Laboratories, West Grove, PA), and Alexa Fluor 546-conjugated anti-rabbit IgG (Invitrogen) were used as secondary Abs. Cell nuclei were visualized using TO-PRO-3 (Invitrogen). The sections were examined using a STELLARIS (Leica, Wetzlar, Germany) instrument. More than four images were captured from the vascular area of each sample and analyzed using ImageJ software for quantitative measurements.

### Cell preparation and flow cytometric analysis

Flow cytometry and cell isolation were performed as described previously (Hu *et al*. 2021). Fluorescently labeled anti-CD44, -CD133 mAbs (BioLegend) were used. The stained cells were sorted using FACSAria (BD Biosciences) or Sonysh800 (Sony, Tokyo, Japan) and analyzed using FlowJo software (Tree Star Software, San Carlos, CA).

### Quantitative reverse transcription PCR (qRT-PCR)

Total RNA was extracted from cells using RNeasy-plus mini kits (Qiagen, Hilden, Germany) and reverse-transcribed using the PrimeScript RT reagent kit (Takara, Tokyo, Japan). Real-time PCR was performed using TB Green Premix Ex Taq II (Takara) on an Mx3000p QPCR system (Agilent, Santa Clara, CA). The primers used for PCR are as follows: mouse Sfrp1, sense 5′-CAG CTT TTG AAC TGG CCA CC-3′, anti-sense 5′- CCT TGC CTG GCA TCC TTG TA-3′; mouse Oct4, sense 5′- TGT TCA GCC AGA CCA CCA TC-3′, anti-sense 5′-GCT TCC TCC ACC CAC TTC TC-3′; mouse Abcg2, sense 5′- CTC ACC TTA CTG GCT TCC GG -3′, anti-sense 5′-ATC CGC AGG GTT GTT GTA GG-3′; mouse Sox2, sense 5′-ACA ACT CCA TGA CCA GCT CG-3′, anti-sense 5′-ACT TGA CCA CAG AGC CCA TG-3′; mouse Bmi1, sense 5′-GAC TCT GGG AGT GAC AAG GC-3′, anti-sense 5′-GTG AGG GAA CTG TGG GTG AG-3′; mouse Ssea1, sense 5′-ACA TCA CCG AGA AGC TGT GG-3′, anti-sense 5′-GCA CGA AGC GCT CAT AGT TG-3′; mouse Ccnd1, sense 5′-CCC TGG AGC CCT TGA AGA AG-3′, anti-sense 5′-AGA TGC ACA ACT TCT CGG CA-3′; mouse Axin2, sense 5′-CCT GAC CAA ACA GAC GAC GA-3′, anti-sense 5′-GCT TCT GCC TCG ATC TCC TC-3′; mouse Lef1, sense 5′-GGC ATG AGG TGG TC AGA CAA-3′, anti-sense 5′-TTG TTG TAC AGG CCT CCG TC-3′; mouse GAPDH, sense 5′-TGG CAA AGT GGA GAT TGT TGC C-3′, anti-sense 5′-AAG ATG GTG ATG GGC TTC CCG-3′. The results were normalized to those of GAPDH using the comparative threshold cycle method.

### Statistics analysis

The data are presented as the mean ± SEM. GraphPad Prism9 software was used for statistical analysis. Data were analyzed using a* t*-test. The level of statistical significance was set at *p* < 0.05 (*p < 0.05, **p < 0.01).

## Results

### Sfrp1 is expressed in a part of tumor ECs

**Figure 1.**
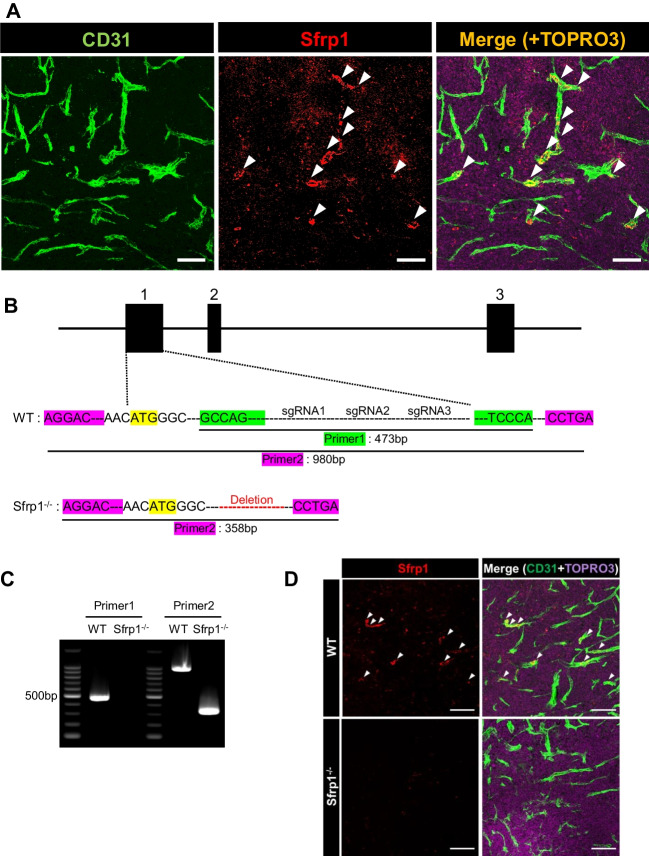
Sfrp1 is expressed in a part of tumor ECs. Immunofluorescence staining of CD31 (*green*), Sfrp1 (*red*, *arrowhead*), and TO-PRO-3 (*purple*) in the frozen section of LLC tumors from WT mice (*A*). *Scale bar* = 100 µm. CRISPR/Cas9-mediated gene engineering strategy for *Sfrp1* and schematic presentation of the Sfrp1 strategy (*B*). PCR analysis of DNA from the tail of Sfrp1 KO and WT mice (*C*). Immunofluorescence staining of CD31 (*green*), Sfrp1 (*red*, *arrowhead*), and TO-PRO-3 (*purple*) in frozen sections of LLC tumors from WT and Sfrp1 KO mice (*D*). *Scale bar* = 100 µm.

We previously reported that Sfrp1 is expressed in fetal apelin receptor (APJ)–positive veins and is involved in arteriovenous alignment (Kidoya *et al*. 2015). To determine the localization of Sfrp1, an angiocrine factor critical for angiogenesis, in tumor tissues, we evaluated its expression. We performed immunostaining to determine the distribution of Sfrp1 within the tumors. Immunostaining revealed that Sfrp1 was expressed in a subset of CD31^+^ vascular ECs (Fig. 1*A*). Positive Sfrp1 signals were not detected in the area of the mouse LLC cancer cells. Further analysis using ImageJ revealed that approximately 8% of CD31^+^ cells expressed Sfrp1. These results suggest that Sfrp1 is expressed in a fraction of the vascular ECs in tumor tissues.

### Generation of *Sfrp1* KO mice

To investigate the effect of Sfrp1 on tumor tissues, the CRISPR/Cas9 gene editing system was used to introduce a deletion into exon 1 of Sfrp1 (Fig. 1*B*). Sanger sequencing (details shown in Supplementary Fig. 1) and PCR were performed to confirm the deletion of Sfrp1 (Fig. 1*C*). We confirmed the absence of Sfrp1 protein expression in LLC tumors of Sfrp1 KO mice via immunostaining (Fig. 1*D*).

### Sfrp1 is required for tumor growth

**Figure 2.**
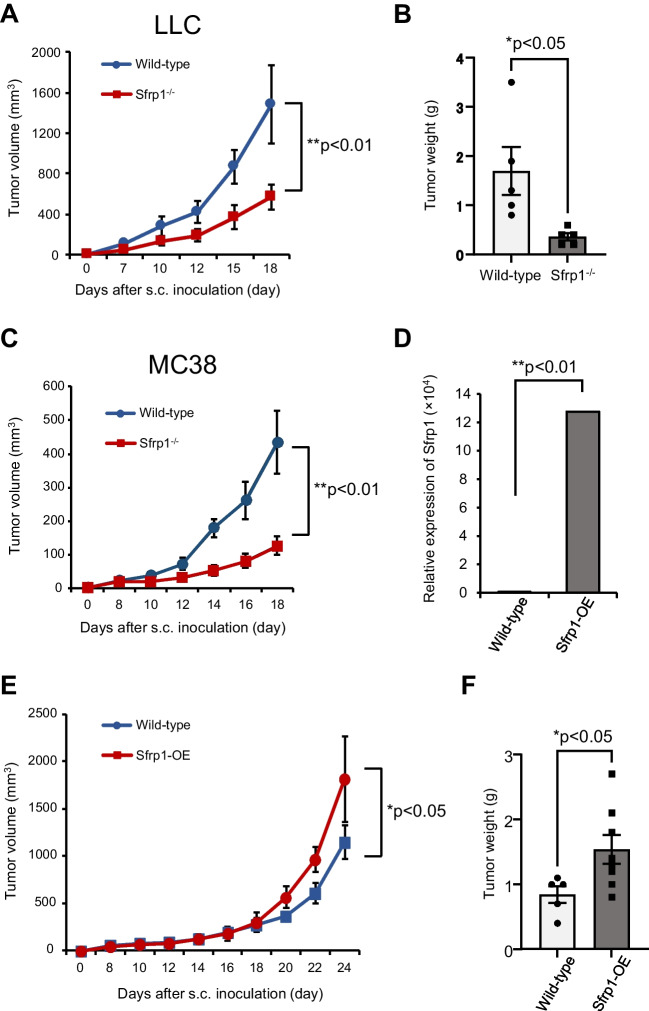
Sfrp1 is required for tumor progression. Tumor growth curves and tumor weight of LLC cells subcutaneously (s.c.) inoculated into WT or Sfrp1 KO mice (*n* = 7 for each group) (*A*, *B*). Tumor growth curves of MC38 cells s.c. inoculated into WT or Sfrp1 KO mice (*n* = 7 for each group) (*C*). Sfrp1 expression was evaluated via qRT-PCR and normalized against that of GAPDH (*D*). Tumor growth curves and tumor weight of Sfrp1-overexpressing LLC cells or LLC cells s.c. inoculated into WT mice (*n* = 7 for each group) (*E*, *F*). *Error bars* indicate ± SEM. *p < 0.05. **p < 0.01.

To examine whether Sfrp1 in the tumor environment affected tumor growth, we inoculated LLC cells into WT and Sfrp1^−/−^ (Sfrp1 KO) mice and evaluated their growth. Compared to the WT group, tumors in Sfrp1 KO mice showed reduced tumor growth (Fig. 2*A*, *p* < 0.01). In addition, the tumor weight was significantly lower in Sfrp1 KO mice than in WT mice (Fig. 2*B*, *p* < 0.05). A similar transplantation experiment was performed using MC38 cells, and tumor growth was suppressed in Sfrp1 KO mice compared with that in WT mice (Fig. 2*C*, *p* < 0.01). To further examine the effect of Sfrp1 on tumor growth in tumor tissues, LLC cells overexpressing Sfrp1 were generated using lipofectamine. Real-time PCR confirmed that the expression of Sfrp1 was significantly upregulated (Fig. 2*D*, *p* < 0.01). Using these Sfrp1-overexpressing cells, we performed tumor transplantation experiments and found that tumor growth was promoted in Sfrp1-overexpressing cells, and tumor weight was markedly increased (Fig. 2*E*–*F*, *p* < 0.05). These data suggest that Sfrp1 is required for the process of tumor growth in tumor tissues.

### Sfrp1 deficient tumors fail to maintain CSCs in the late stages of tumor growth

**Figure 3.**
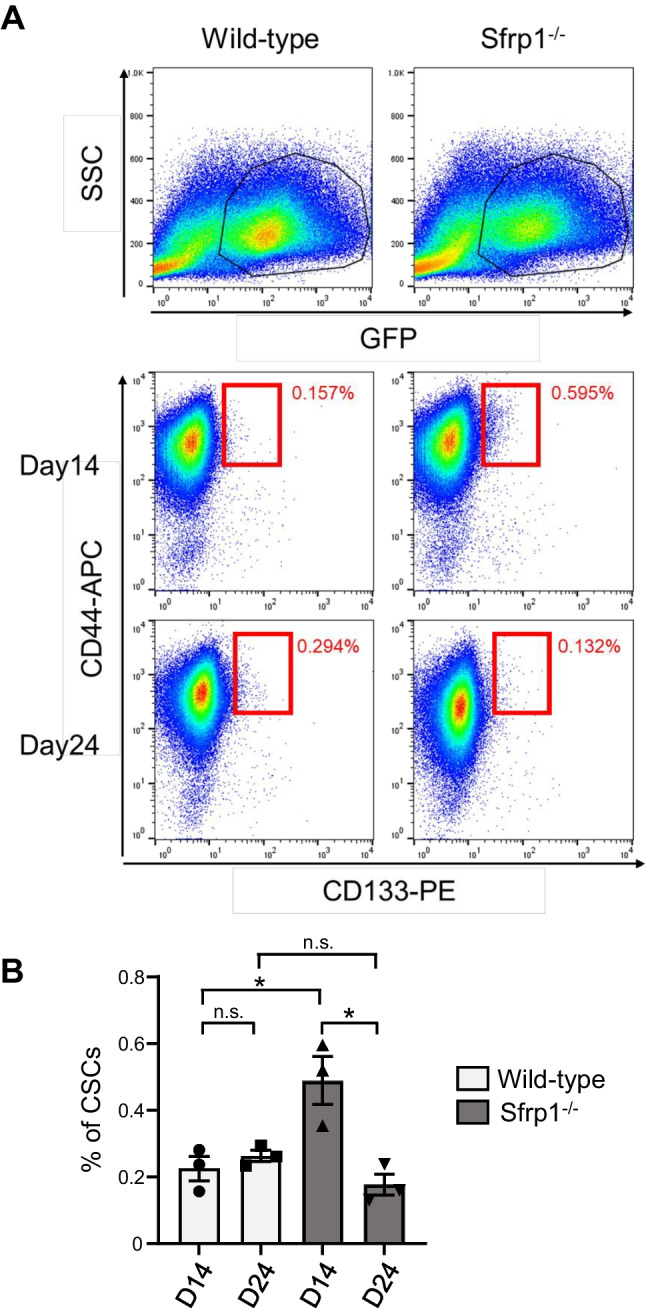
Sfrp1 deficient tumors have a reduced proportion of CSCs in the late stages of tumor growth. Flow cytometry was used to isolate GFP^+^ CD44^+^ CD133^+^ LLC cells from WT and Sfrp1 KO mice bearing tumors at days 14 and 24 (*A*). Quantification of GFP^+^ CD44^+^ CD133^+^ CSCs (*n* = 3) (*B*). *Error bars* indicate ± SEM. *p < 0.05.

Some CSCs in tumor tissues exist in a state of arrested cell proliferation, and their presence contributes to tumor growth and resistance to anticancer drugs. Tumor blood vessels are a niche for CSCs. Therefore, we examined the effect of Sfrp1, which is expressed in some tumor ECs, on CSCs. To test the effect of Sfrp1 on tumor tissues, we determined the percentage of CD44^+^ CD133^+^ tumor cells in Sfrp1 KO and WT mice. Initially, we generated cells expressing GFP in LLC cells and transplanted them subcutaneously. On examining the percentage of GFP^+^ CD44^+^ CD133^+^ CSCs via FACS 14 d after transplantation, a higher percentage of CSCs was noted in Sfrp1 KO mice (Fig. 3A–B, *p* < 0.05). In addition, tumor retrieval 24 d after transplantation showed a drastic decrease in the percentage of CSCs in Sfrp1 KO mice. These results suggest that Sfrp1 KO mice exhibit an abundance of CSCs in the early stages of tumor growth; however, as tumor growth progresses, the proportion of CSCs decreases significantly, and tumor growth is suppressed.

### Sfrp1 does not affect the structure of tumor blood vessels

**Figure 4.**
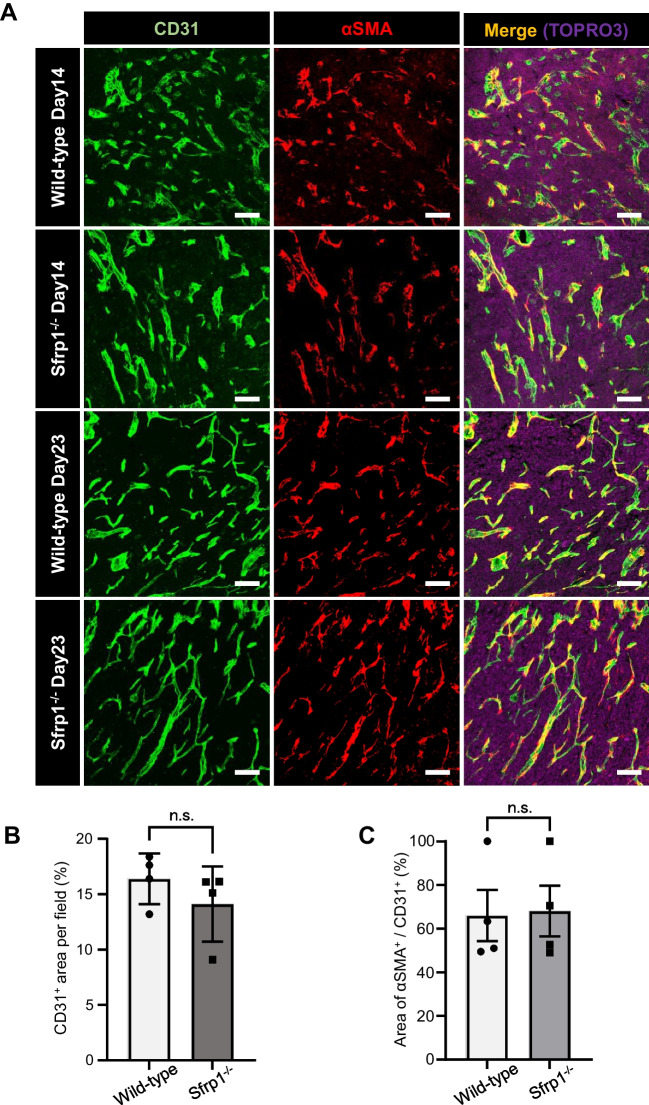
Sfrp1 does not affect the structure of tumor blood vessels in tumor tissues. Immunostaining of sections from LLC tumor-bearing WT or Sfrp1 KO mice 14 or 23 d after implantation. ECs were stained with anti-CD31 antibody (green), and perivascular cells were stained with anti-αSMA antibody (red) (*A*). *Scale bar* = 100 µm. Quantification of the CD31^+^ area per field in Sfrp1 KO and WT mice (*n* = 4) (*B*). Quantification of the area of αSMA^+^ per CD31^+^ area in Sfrp1 KO and WT mice (*n* = 4) (*C*). *Error bars* indicate ± SEM.

We previously reported that Sfrp1 affects the vascular structure of fetal skin (Kidoya *et al*. 2015). Therefore, immunostaining was performed to examine the effect of Sfrp1 on the structure of blood vessels in tumor tissues. LLC cells were transplanted into Sfrp1 KO and WT mice, and tumors were collected 14 and 23 d later. Collected tissues were stained with the vascular EC marker CD31 and the vascular pericyte marker αSMA. A comparison of the tumor vasculature between Sfrp1 KO and WT mice showed no significant differences. Tumor growth was suppressed 23 d after transplantation in Sfrp1 KO mice, but no changes were observed in the structure of tumor vasculature (Fig. 4*A*). The CD31^+^ vascular area was measured and compared between the WT and KO mice, but no changes were observed (Fig. 4*B*). The areas of αSMA^+^/CD31^+^ were also analyzed, showing no significant difference between WT and KO mice (Fig. 4*C*). These results suggest that the effect of Sfrp1 on tumor growth is unrelated to the structure tumor vasculature.

### Sfrp1 contributes to CSC maintenance via Wnt signaling

**Figure 5.**
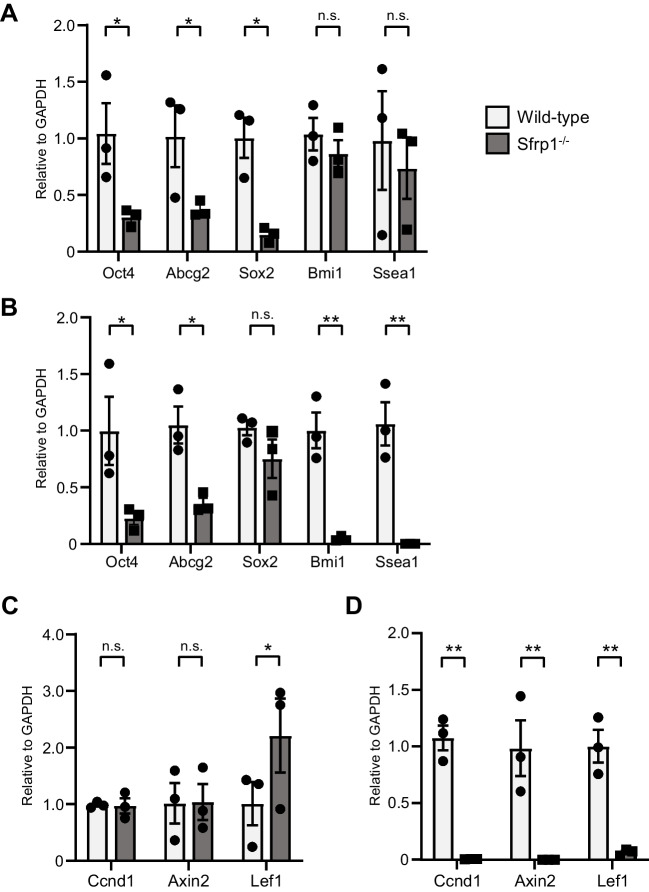
Examination of Wnt signaling by Sfrp1 in tumor tissues. qRT-PCR analysis of mRNA expression of CSC markers (Oct4, Abcg2, Sox2, Bmi1, and Ssea1) in GFP^+^ CD44^+^ LLC cells collected from WT or Sfrp1 KO mice 14 d after transplantation via FACS, relative to GAPDH expression (*A*). Twenty-three d after transplantation (*B*). qRT-PCR analysis of mRNA expression of Wnt signaling target genes (Ccnd1, Axin2, and Lef1) relative to that of GAPDH (*n* = 3). GFP^+^ CD44^+^ LLC cells collected from WT or Sfrp1 KO mice 14 d after transplantation (*C*). Twenty-three d after transplantation (*D*). *Error bars* indicate ± SEM. ** shows *p* < 0.01, * shows *p* < 0.05, statistical significance; n.s., not significant.

The mechanism of action of Sfrp1 in tumor tissues is not well understood. It has been reported that Sfrp1 binds to Frizzled, a Wnt signaling receptor, and competitively inhibits Wnt signaling. However, the mechanism by which Sfrp1 acts directly on Frizzled remains unknown (Zhan *et al*. 2017). Therefore, we focused on Wnt signaling in CCs during the early and late stages of tumorigenesis. Flow cytometry was used to collect GFP^+^ CD44^+^ CCs 14 d after tumor cell transplantation, and qRT-PCR was used to examine the expression of CSC markers (Oct4, Abcg2, Sox2, Bmi1, and Ssea1). Sfrp1-deficient cancer tissues showed decreased expression of CSC markers in CCs (Fig. 5*A*). Moreover, GFP^+^ CD44^+^ CCs were obtained and investigated 23 d after tumor transplantation. After 14 and 23 d, the expression of most CSC markers was significantly reduced in Sfrp1 KO mice (Fig. 5*B*). Next, we examined the expression of Wnt signaling target genes (Ccnd1, Axin2, and Lef1) in GFP^+^ CD44^+^ CCs at 14 and 23 d after inoculation. In the early stages of tumor transplantation, the expression of Wnt signaling target genes was observed in the CCs of Sfrp1 KO mice. On day 14, there was no significant decrease in the expression of Wnt signaling target genes in Sfrp1 KO mice compared with that in WT mice. In contrast, the expression of Wnt signaling target genes was significantly decreased in the late stage after tumor inoculation in the CCs of Sfrp1 KO mice (Fig. 5*C*–*D*).

## Discussion

In this study, we demonstrated that Sfrp1, an angiocrine factor produced by tumor blood vessels in the tumor environment, is involved in tumor growth. Analysis using WT and Sfrp1 KO tumor model mice suggested that Sfrp1 is involved in maintaining the stemness of CCs without affecting tumor vasculature. Although the detailed effects of Sfrp1 in tumor tissues remain unknown, we demonstrated that Sfrp1 contributes to tumor growth by regulating CSC proliferation.

Sfrp1 reportedly serves as an antagonist by binding to Wnt ligands. However, our findings suggest that it is not an antagonist but a regulator of Wnt signaling in the tumor environment. Another possible signaling mechanism is the direct binding of Sfrp1 to Frizzled receptors. Preliminary studies have examined Frizzled expression in CSCs, revealing significant expression of Fzd 1, 4, and 5. It is possible that these receptor-mediated signals support stem cell maintenance mechanisms. As for Fzd expression in CCs, Fzd1 expression showed a declining rate compared to that in CSCs (data not shown). However, the difference in sensitivity to the receptor might have influenced the results of this study, which should be clarified in future studies.

Our results indicate that Sfrp1 may regulate CSC self-renewal and transient malignant growth and act to maintain a dormant state (Fig. 3). In the absence of Sfrp1 in the tumor environment, CSC growth increases in the early stages of tumor growth but decreases in the late stages, which may attenuate tumor progression. The transcript levels of canonical Wnt signaling targets in tumor tissues of Sfrp1-deficient mice were similar to those in WT mice in the early phase of tumor development but were markedly reduced in the late phase (Fig. 5). However, the proportion of CSCs in tumor tissue increased in Sfrp1-deficient mice in the early phase (Fig. 3). This suggests that canonical Wnt signaling may not be involved in the maintenance of CSC dormancy during the early phase of tumor development. Nonetheless, further analysis is needed to elucidate the mechanism of regulatory signaling by Sfrp1 in CSCs.

The results of this study suggest that Sfrp1 is expressed in a portion of tumor vessels in the CSC niche, indicating that the blood vessels within the tumor are not homogeneous but rather heterogeneous. Such a vascular niche has been previously proposed in glioblastomas, indicating that CSCs reside near blood vessels and are maintained by Notch signaling (Calabrese *et al*. 2007). A stem cell maintenance mechanism in response to Wnt signaling has also been proposed in colorectal cancer. GLI1-positive mesenchymal cells in colorectal cancer tissues secrete Wnt, aiding in the maintenance and promotion of stem cell self-renewal (Degirmenci *et al*. 2018). This report describes the mechanism of stem cell maintenance by mesenchymal cells. However, the mechanism of CSC niche formation by Sfrp1-positive ECs observed in the present study was also regulated by Wnt signaling and is similarly relevant.

It has also been reported that Sfrp1 promotes vascular maturation by enhancing the number of mesenchymal stem cells around neovascular vessels (Dufourcq *et al*. 2008). In contrast, the present study showed that Sfrp1 did not affect tumor angiogenesis or vascular structure. Sfrp1 plays a vital role in the formation of the dense structure of arteries and veins during fetal development (Kidoya *et al*. 2015). While Sfrp1 was expressed in many APJ-positive veins during this period, only approximately 8% of tumor vessels showed Sfrp1 expression. Perhaps changes in vascular structure could be observed at the time of tumor implantation or in the early stages of angiogenesis; however, we did not analyze this because there was no significant difference in tumor size at that time.

Several cancer therapies have been developed; however, developing anticancer drugs that directly target proliferating cells has been challenging due to significant side effects and the ability of CSC to develop drug resistance. Further, the development of anticancer agents targeting blood vessels has been explored, but these compounds have exhibited lower-than-expected efficacy and are not discriminatory, affecting blood vessels throughout the body, resulting in significant side effects (Ebos and Kerbel 2011; Liu *et al*. 2023). It has recently been proposed that ECs that constitute blood vessels do not represent a uniform cell population, which applies to tumor vessels as well. Therefore, targeting specialized vascular ECs involved in niche formation is expected to destroy the CSC niche, establishing a method for inhibiting tumor growth with minimal side effects.

## Conclusions

Sfrp1 in tumor tissues is expressed in a subset of vascular ECs. Furthermore, Sfrp1 may promote tumor growth by contributing to the maintenance of CSCs via Wnt signaling.

## Supplementary Information

Below is the link to the electronic supplementary material.

Supplementary file1 (PDF 9.40 KB)

## Data Availability

The data that support the findings of this study are available from the corresponding author on reasonable request.
