# Supplementary material for: Tumor endothelial cell-derived Sfrp1 supports the maintenance of cancer stem cells via Wnt signaling
Source: In Vitro Cell Dev Biol Anim. 2024 Apr 16;60(10):1123–31. doi: 10.1007/s11626-024-00899-y (PMC11655579; doi:10.1007/s11626-024-00899-y)
Supplement: Supplementary file 1 — Supplementary file1 (PDF 9.40 KB) [file 11626_2024_899_MOESM1_ESM.pdf]

Supplementary Figure 1

WT mice

AGGACCCCATCGATCGGAGACCCGGGGAGCAGCGCGCAGCCGCCGAGCCGGACGGGGCCC
GGCACTGCGCCTTTGTCCCCGGAGGCTCCGGGAAGTTTGCAGCGGGACGCGCGCGTGAAGG
CAGCGTGGGCAGCCCCGACGTCGCCGAGCAACATGGGCGTCGGGCGCAGCGCGCGGGGTCG
CGGCGGGGCCGCCTCGGGAGTGCTGCTGGCGTTGGCCGCCGCTCTGCTGGCCGCGGGTTCGG
CCAGCGAGTACGACTACGTGAGCTTCCAGTCCGACATCGGCTCGTATCAGAGCGGGCGCTTC
TACACCAAGCCCCCGCAGTGCGTGGACATCCCGGTGGACCTGAGGCTGTGCCACAACGTGG
GCTACAAGAAGATGGTGCTGCCCAACCTGCTGGAGCACGAGACCATGGCAGAGGTGAAGCA
GCAGGCCAGCAGCTGGGTGCCGCTGCTCAACAAGAACTGCCACATGGGCACCCAGGTCTTC
CTCTGTTCGCTCTTCGCGCCCGTCTGTCTGGACCGGCCCATCTACCCGTGTCGCTGGCTCTGC
GAGGCCGTGCGCGACTCGTGCGAGCCGGTCATGCAGTTCTTCGGCTTCTACTGGCCCGAGAT
GCTCAAATGTGACAAGTTCCCCGAGGGCGACGTCTGCATCGCCATGACCCCGCCCAATACCA
CGGAAGCCTCTAAGCCCCAAGGTAAGGGTATGCCTTCCCACGCCCGCGAACCCCCGCGCCCA
TCCGCACTGGCCCAGGGCTGTAGAGGGGGAAAGCTCAGGAGCCGCGCACCGCAACCCGCAA
GGTGTGCAGACCCGATCCCTAAAGCTTCTGGGAGGCCCAGGCATCGCTTGGCGCTTCTCCCG
CTCCAGGTACTCCCTGATGGCTGGTGAGCCTCGGCCGTGGGGGGGGGGGGTTGGGGGGG
CGAGGGGAGACCTGGAGCTGCTCTTCCTAAGCTCCAGTCTGGCGTTTTCCATACCTGA

Sfrp1 KO mice

AGGACCCCATCGATCGGAGACCCGGGGAGCAGCGCGCAGCCGCCGAGCCGGACGGGGCCC
GGCACTGCGCCTTTGTCCCCGGAGGCTCCGGGAAGTTTGCAGCGGGACGCGCGCGTGAAGG
CAGCGTGGGCAGCCCCGACGTCGCCGAGCAACATGGGCGTCGGGCGCAGCGCGCGGGGT
CGCGGCGGGGCCGCCTCGGGAGTGCTCCGCTCGTATCAGACCAAGCCCCCGCACTCCCG
CTCCAGGTACTCCCTGATGGCTGGTGAGCCTCGGCCGTGGGGGGGGGGGGTTGGGGGG
GGCGAGGGGAGACCTGGAGCTGCTCTTCCTAAGCTCCAGTCTGGCGTTTTCCATACCTGA

gRNA1:ACATCGGCTCGTATCAGAGC_GGG
gRNA2:CTGAGGCTGTGCCACAACGT_GGG
gRNA3:CAAATGTGACAAGTTCCCCG_AGG

Genotyping primer1 WT473bp, KO-
Genotyping primer2 WT980bp, KO358bp
